# Voice Quality in Individuals With Acquired Immunodeficiency Syndrome

**DOI:** 10.1111/1460-6984.70309

**Published:** 2026-09-02

**Authors:** Vanessa Medina, Andrea Baldi de Freitas, Luiz Carlos Rusilo, Jean Carlo Gorinchteyn, Zuleica Antônia Camargo

**Affiliations:** ^1^ Research Group on Speech Studies (GeFALA), Laboratory on Acoustic Analysis and Cognition (LIAAC), Postgraduate Studies Programme in Applied Linguistics and Language Studies Pontifical Catholic University of São Paulo (PUC ‐ SP) São Paulo São Paulo Brazil; ^2^ Science and Technology Institute Universidade Federal de Alfenas (UNIFAL) Poços de Caldas Minas Gerais Brazil; ^3^ Emilio Ribas Infectology Institute Secretaria de Saúde do Estado de São Paulo São Paulo São Paulo Brazil; ^4^ Faculty of Philosophy, Communication, Languages and Arts (FAFICLA), Department of Language Sciences and Philosophy, Postgraduate Studies Programme in Applied Linguistics and Language Studies (PEPG‐LAEL) Pontifical Catholic University of São Paulo (PUC‐SP) São Paulo São Paulo Brazil

**Keywords:** acquired immunodeficiency syndrome, auditory perception, evaluation, larynx, voice acoustics

## Abstract

**Background:**

Speech and voice disorders are frequently observed in individuals with acquired immunodeficiency syndrome (AIDS). However, the literature lacks detailed descriptions of how perceptual and acoustic characteristics manifest in the voice quality profiles of this population.

**Aim:**

To characterize the voice quality profile of older adults living with AIDS by describing the relationship between perceptual and acoustic voice measures.

**Methods:**

The research group (RG) comprised 20 individuals with AIDS, aged 50–60 years, all presenting with lipodystrophy, metabolic conditions including dyslipidemia, and 2–9 years of antiretroviral therapy. The control group (CG) comprised 9 age‐matched individuals with metabolic conditions, including dyslipidemia, but without AIDS or antiretroviral therapy. The corpus included semi‐spontaneous speech samples and repetitions of three key sentences designed for the perceptual evaluation of voice quality settings (VQS) across the vocal tract, muscular tension, and phonatory domains. For the acoustic analysis, data were processed using a script in the Praat software, which automatically extracted measures of fundamental frequency (f0), intensity, spectral slope, and the long‐term average spectrum (LTAS). A preliminary statistical analysis was performed to establish the basis for presenting the results. Among the sociophonetic factors examined, gender emerged as the most influential variable and was included in the multivariate statistical approach.

**Results:**

The results indicated a significant prevalence of vocal tract VQS in the RG, including a retracted and lowered tongue body, pharyngeal constriction, and vocal tract hyperfunction, demonstrating strong discriminative power for classifying RG (98.7%) and CG (98.3%) samples. Acoustic measures showed a lower correlation factor (R^2^: 36.5%) for distinguishing the RG (77.4%) and CG (77.5%) samples. Regarding the relationship between acoustic measures and perceived voice qualities, f0 (particularly for females), LTAS (especially for males), and spectral slope were found to be relevant.

**Conclusions:**

Vocal tract and tension VQS, together with correlated spectral measures, were relevant in the RG. These findings may reflect the combined functional consequences of long‐term antiretroviral therapy, opportunistic diseases, lipodystrophy, and associated metabolic alterations. The results highlight the need for further studies to support the future development of preventive, monitoring, and rehabilitative speech‐language pathology strategies during the long‐term follow‐up of individuals living with AIDS.

**WHAT THIS PAPER ADDS:**

*What is already known on this subject*

- Research suggests a high prevalence of speech and voice disorders among patients with acquired immunodeficiency syndrome (AIDS). However, the relationship between perceived and acoustic voice quality in this population remains unexplored.

*What this study adds to existing knowledge*

- Adjustments in vocal tract and tension‐related voice quality, along with correlated spectral measures, were found to be significant in RG. These findings may relate to reported effects of lipodystrophy on the vocal tract and upper respiratory infections from opportunistic diseases.

*What are the clinical implications of this study?*

- The findings of this study may be linked to the effects of lipodystrophy on the vocal tract in AIDS patients, as well as upper airway weakness due to opportunistic diseases. This underscores the need to develop therapeutic strategies for ongoing care in the AIDS population.

## 1 Introduction

Acquired Immunodeficiency Syndrome (AIDS) is characterized by the emergence of multiple, successive, or simultaneous opportunistic diseases resulting from the weakening of the body's defences. These diseases, which stem from immunodeficiency, destroy CD4+ lymphocytes responsible for the body's immune response. A compromised immune system facilitates opportunistic infections caused by viruses, bacteria, fungi, and protozoa (Jordan et al. 2020; Siedner and Triant 2019). Among the consequences of these infections, speech and voice disorders, particularly laryngeal disorders, have been documented (Badji et al. 2022; de Souza Fonseca et al. 2022; Piersiala et al. 2022).

Within this context, this study sought to examine voice quality in older people with AIDS from a phonetic perspective, focusing on perceptual and acoustic counterparts. Although AIDS has been studied since 1981, the prevention and management of complications arising from opportunistic diseases and medication side effects remain challenging (Dominguez Filho et al. 2021; Furrer 2016; Obeagu and Nwosu 2019). Moreover, the long‐term effects of antiretroviral therapy are not yet fully understood, and research regarding changes in oral communication within this population remains limited.

In Brazil, AIDS remains an important public health concern. According to the Ministry of Health (Ministério da Saúde 2025), 1,165,533 cases of AIDS were reported between 1980 and September 2025, with 402,300 deaths recorded between 1980 and 2024. The Brazilian Unified Health System guarantees universal and free access to antiretroviral therapy and long‐term follow‐up, contributing to increased survival and shifting care towards comorbidity management, preservation of functionality, and improved quality of life (Alves et al. 2023; Chawla et al. 2018; DST/AIDS 2018).

Alterations affecting the speech mechanisms of individuals with AIDS also impact the respiratory system (lungs, bronchi, and trachea), which supplies the air flow for speech; the phonatory system (larynx and vibrating vocal folds); and the articulatory and resonant structures, including the vocal tract and subpharyngeal cavities—oral, nasal, and pharyngeal spaces. Oral communication disorders have also been attributed to neurological complications associated with AIDS (Brandão Canut et al. 2021; Laver et al. 1981).

Other frequent symptoms and manifestations, in both adult populations, include middle ear pathology, conductive and sensorineural hearing loss, changes in voice quality, viral and fungal infections of the ear, nose, throat, and mouth, hypopharyngeal and laryngeal dysfunction due to tumours and infections, apathy, forgetfulness, cognitive decline, and depression (Askari et al. 2021; Haase et al. 2021; Ivanova et al. 2023; McLaurin et al. 2021; Ripamonti and Clerici 2021; Shanmuga Ashok and Rajendran 2020).

Although previous studies have suggested these changes, they have not comprehensively investigated alterations in the structures involved in voice production among people with AIDS. It is crucial to assess these changes in oral communication to identify effective intervention strategies for this population, whose specific needs make them particularly dependent on innovations in the treatment of the syndrome, especially regarding prolonged therapy and increased survival rates (Lomelí‐Martínez et al. 2022). There is evidence indicating a predisposition to speech production disorders within this group (Badji et al. 2022; Brandão Canut et al. 2021; Fisicaro et al. 2023; Piersiala et al. 2022).

Integrating speech technologies enables detailed characterization of the physiological, perceptual, and acoustic features of speech and voice in people with AIDS. These technologies may support the identification of speech and voice biomarkers across different stages of the disease, from diagnosis to short‐ and long‐term follow‐up (Barche et al. 2020; Brandão Canut et al. 2021; Chandrashekar et al. 2019).

The phonetic approach is appropriate as it describes the specificities of voice production through voice quality settings (VQS), encompassing adjustments in the phonatory, vocal tract (subpharyngeal), and muscular tension domains of the speech system. By integrating these dimensions, VQS provides a comprehensive characterization of vocal tract function rather than focusing solely on isolated laryngeal features. The Vocal Profile Analysis Scheme (Laver et al. 1981) and its Brazilian Portuguese adaptation (VPAS‐PB) (Camargo and Madureira 2008) were therefore adopted. Probable alterations in the speech and vocal systems of people with AIDS have been attributed to possible hypotonia of the orofacial musculature and vocal folds, resulting from lipodystrophy (Lamesa 2024).

Considering the clinical heterogeneity of older adults living with AIDS, including age‐related changes, long‐term antiretroviral therapy, and clinical manifestations that may affect the speech and voice production mechanisms, descriptive studies are essential to characterize voice quality patterns in this population (Chawla et al. 2018; Lamesa 2024). Individuals affected by the disease may present laryngeal conditions such as chronic, fungal, and ulcerative laryngitis, as well as laryngeal cancer, potentially directly affecting vocal production and voice quality (Piersiala et al. 2022; Vazquez 2000). Despite these manifestations, the voice quality profile of older adults living with AIDS has not yet been comprehensively characterized. Hence, perceptual‐auditory and acoustic parameters of speech and voice may function as potential biomarkers of subtle functional alterations related to laryngeal behaviour, pneumophonoarticulatory coordination, neuromotor speech control, and swallowing‐related dynamics (Barche et al. 2020; Camargo and Madureira 2009; Chandrashekar et al. 2020).

Thus, descriptive studies are intended to document vocal characteristics and characterize functional alterations of the vocal tract (Camargo and Madureira 2008; Laver et al. 1981). Perceptual‐auditory and acoustic findings may provide clinically relevant information on the anatomical and physiological adjustments underlying communication and upper aerodigestive functions, including swallowing (Brandão Canut et al. 2021; Camargo and Madureira 2009). These findings may serve as early clinical indicators of speech and language pathology manifestations, supporting longitudinal monitoring and the development of preventive and rehabilitative strategies.

Therefore, this study aimed to address perceptual and acoustic correlates of voice quality as biomarkers indicating long‐term sequelae of antiretroviral drug use and the potential need for speech and language therapy to mitigate communication disorders that can impact quality of life. This study posited the hypothesis of specific voice quality findings in individuals with AIDS, supported the investigation of such correlates, related the disease to sustained antiretroviral therapy, and proposed the description of voice quality within the framework of speech biomarkers. By describing the relationship between perceptual and acoustic data on voice quality, this study aimed to advance understanding of the acoustic characteristics associated with AIDS.

## 2 Methods

This project, grounded in phonetics, is a cross‐sectional study employing a retrospective, descriptive design based on perceptual and acoustic analyses. All participants signed the Free and Informed Consent Form, indicating their agreement to the use of their information strictly for scientific purposes and to the inclusion of the collected samples in the research database.

The studied groups comprised 29 adult individuals of both genders, assigned to two groups:

The research group (RG) comprised 20 individuals with AIDS (10 men and 10 women) undergoing antiretroviral treatment. Participants were recruited from the Older Adult Outpatient Clinic at a tertiary reference service linked to the Brazilian Unified Health System. The inclusion criteria for the RG were selfg‐referral, confirmation of an AIDS diagnosis (provided by the research institution), antiretroviral treatment for at least 2 years, presence of lipodystrophy and metabolic conditions (including dyslipidaemia), native proficiency in Brazilian Portuguese, and age between 50 and 60 years. The exclusion criterion was a self‐reported history of speech and voice disorders.

The control group (CG) included 9 individuals (5 women and 4 men), matched in age to the RG. Participants were recruited from the same healthcare institution. The inclusion criteria for the CG were absence of AIDS (as documented by the institution), absence of antiretroviral treatment, presence of metabolic conditions (including dyslipidaemia), native proficiency in Brazilian Portuguese, and age between 50 and 60 years. The exclusion criterion was a self‐reported history of speech and voice disorders. The inclusion of a CG with metabolic conditions without AIDS or exposure to antiretroviral therapy was intended to support comparison while considering metabolic alterations as part of the clinical profile of both groups.

### 2.1 Data Collection Procedures

The samples were collected in a soundproof room to control internal and external noise that could affect the quality of the recordings and analysis. To reduce the room's noise level to below 50 dBNP, three measurements were taken at different points using an Instrutherm DEC‐460 digital sound pressure meter with a calibrator (94 dB at 1 kHz), at distances of 50 cm from each other and 1.5 m from the walls, windows, doors, and floor. The values obtained were averaged logarithmically.

Audio samples were recorded using Sound Forge software at a sampling frequency of 22,050 Hz, 16 bits, WAV format, mono channel, installed on an HP Pavilion dv6000 laptop (Intel Core2Duo T7250 processor [2 GHz, 2 MB L2 cache, 800 MHz FSB], 260 MB RAM), Realtek High Definition Audio sound card, Creative Labs SB1090 USB Sound Blaster X‐Fi Surround 5.1 external sound card, Eurorack UB502 preamplifier analogue audio mixer, running the Windows Vista Home Premium operating system, and a Shure WH20 XLR unidirectional professional headset microphone.

Before each recording, the acoustic signal was calibrated to account for individual differences in intensity. For this procedure, the microphone was positioned at an angle of 45–90°, at less than 10 cm (approximately 3–4 cm from the speaker's mouth). In this setup, the speaker was asked to count to a designated number. The recorded acoustic signal was identified using the VU meter option of the Sound Forge software, the mixer's gain was adjusted until the input ranged from −3 to −9 dB, and the gain value was recorded.

**FIGURE 1:**
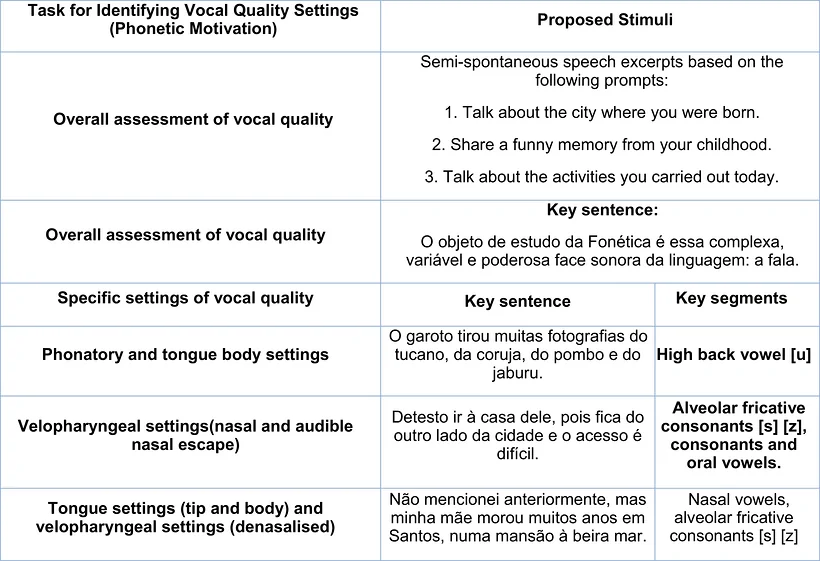
The corpus for the phonetic assessment of voice quality settings based on instructional material developed for application of the VPAS‐PB instrument (Camargo and Madureira 2008). Each task was audio‐recorded to capture semi‐spontaneous speech (prompts 1–3). The carrier sentences were also presented in printed form for reading, in random order, with each sentence repeated four times. Speakers were given the opportunity to read the sentences beforehand and request clarifications from the researcher as needed.

Audio recording sessions were conducted over five months, from June to October 2012. During this period, information about the duration of antiretroviral medication was also collected. The recordings were made from a corpus selected for phonetic motivation to assess voice quality (Figure 1), incorporating semi‐spontaneous speech samples and sentence readings designed for use with the VPAS‐PB (Camargo and Madureira 2008).

Although the data were collected in 2012, they are part of a systematically catalogued and securely stored database. The digital preservation protocol ensured high acoustic fidelity, and the methods and parameters used in the analyses remain valid. Therefore, the scientific relevance and integrity of the dataset have been preserved.

### 2.2 Data Analysis Procedures

Audio recording samples were edited and labelled as semi‐spontaneous speech (SSS) and reading speech (RS) utterances. The SSS excerpts were selected from authorized recordings of spontaneous speech, each with a minimum duration of one minute, to ensure sufficient vocal material for perceptual analysis. In total, approximately 137 min of recordings were collected from RG speakers (individuals with AIDS) and 44 min from CG speakers. These samples were analysed from both perceptual and acoustic perspectives.

#### 2.2.1 Perceptual Analysis of Voice Quality

The VPAS‐PB (Camargo and Madureira 2008), adapted from the original VPAS protocol (Laver 1980), was employed for perceptual analysis. This protocol is based on the characterization of the combination of VQS in phonation, the vocal tract, and muscular tension (vocal tract and larynx). The analysis considered adjustments in the vocal tract (e.g., backed tongue body, lowered tongue body, pharyngeal constriction, labiodentalization, lip extension), laryngeal and phonatory settings (e.g., harsh voice, breathy voice, creaky voice, whisper, raised larynx), and muscular tension (e.g., tense vocal tract, lax vocal tract). For clarity, a complete list of the VQS used, with brief definitions, is provided in Appendix A.

The VPAS‐PB has been gradually incorporated into speech and language therapy practice and applied in various clinical and research contexts. Its applications include studies involving children with cochlear implants (Pessoa‐Almeida et al. 2014), individuals with acromegaly (Baldi 2017), and teachers with voice disorders (Fernandes et al. 2025; Silva et al. 2017), as well as experimental investigations of articulatory adjustments in children with altered lingual frenulum (Camargo and Canton 2020), and analyses exploring the relationship between perceptual and acoustic data (Camargo et al. 2019). The protocol has also been explored in innovative contexts, such as combination with ultrasound imaging (Jesus et al. 2021) and in the perceptual analysis of speech in laryngectomized individuals (Condori et al. 2025). In addition, international studies have highlighted its potential for speaker characterization in forensic phonetics (San Segundo et al. 2019).

This phase involved three judges, specialists in phonetic assessment of vocal quality. The judges were not involved in the study design or data collection. Speech samples were presented in coded format, with no indication of group allocation (RG or CG), ensuring that the judges were blinded to the sample origin during evaluation. Judges used the freely available software Praat and headsets to evaluate the samples individually (Boersma and Weenink 2018).

Each utterance (SSS and RS) was evaluated using the VPAS‐PB. After individual assessments, the three examiners reviewed the results and, in cases of disagreement regarding the nature or degree of any vocal setting, conducted a joint analysis until reaching consensus. Discussions focused on standardizing nomenclature and grading vocal settings, resulting in high agreement.

#### 2.2.2 Acoustic Analysis

Acoustic analysis of the audio samples was conducted using the ExpressionEvaluator script in the freely available Praat software. This script enables the automatic extraction of measures related to the fundamental frequency: f0 (median, interquartile range, 99.5% quantile, and asymmetry), first derivative of f0 (mean, standard deviation, and asymmetry), intensity (mean, standard deviation, and asymmetry), and long‐term average spectrum (LTAS; standard deviation).

#### 2.2.3 Statistical Analysis

A preliminary statistical analysis was performed using discriminant analysis in XLStat (Addinsoft) to identify the principal socio‐phonetic variables that could categorize the data into distinct classes. Distinct values of a set of variables indicate whether a given element belongs to a particular group. Among the variables studied (gender, age group, place of birth, and speaking styles), gender emerged as the most influential. The male and female subgroups exhibited high discriminative power, representing highly distinct classes (100% discriminative power for both perceptual descriptions and acoustic measurements).

Based on these findings, separate statistical treatments were applied to the male and female subgroups for both the RG and the CG. In the statistical analyses conducted, including discriminant analysis, confidence interval construction, and linear regression, hypothesis testing was carried out with the significance level set at 1% to compare perceptual (VPAS‐PB data) and acoustic (Expression Evaluator script data) quality variables between the female and male subgroups of the RG and CG.

## 3 Results

### 3.1 Perceptual Data: Voice Quality Profiles

The perceptual analysis indicated that female speakers in the RG exhibited VQSs characterized by pharyngeal constriction and reduced movement of the tongue and jaw. Additionally, phonatory features such as creaky voice and brief diplophonic episodes were identified. In contrast, the CG demonstrated vocal tract configurations including increased lip extension, retracted tongue tip, and anterior positioning of the tongue body. These patterns were observed based on the confidence intervals calculated for each voice quality setting, with significance set at 1 (Table 1).

**TABLE 1:** Confidence intervals for voice quality settings for the female research and control groups.

Female group	Voice quality settings	No.	Type	IL	SL
Research group	Vocal tract	1	Open jaw and minimized range	0.23	0.49
		2	Backed tongue body	0.32	0.62
		3	Raised tongue body	0.35	0.65
		4	Tongue body—minimized range	0.53	0.89
		5	Pharyngeal constriction	1.43	1.82
		6	Raised larynx	0.92	1.22
	Phonatory	7	Creaky voice	0.69	0.99
		8	Short‐term events—diplophonia	0.07	0.18
Control group	Vocal tract	9	Lips—extensive range	0.13	0.28
		10	Retracted tongue tip	0.11	0.27
		11	Fronted tongue body	0.39	0.73

Abbreviations: IL, inferior limit; SL, superior limit.

The analysis of VQS in the male subgroup revealed consistent differences between the RG and the CG. In the RG, perceptual adjustments were concentrated in the vocal tract, with prominent features including tongue retraction and lowering, as well as pharyngeal constriction. These features were accompanied by signs of imbalanced muscular tension, including a tense vocal tract and a lax larynx. Phonatory configurations included whisper, harsh voice, and short‐term phenomena, such as brief pauses, suggesting irregularities in vocal production.

In contrast, CG participants exhibited vocal patterns more indicative of articulatory stability, characterized by a greater range of lip and jaw movement and minimal evidence of marked phonatory effort. The occurrence of whispery voice in this group was isolated and not accompanied by other significant deviations. These findings suggest a more complex, multifactorial pattern of vocal adjustment in RG individuals, with functional implications (Table 2).

**TABLE 2:** Confidence intervals for voice quality settings for the male research and male control groups.

Male group	Voice quality settings	No.	Type	IL	SL
Research group	Vocal tract	1	Retracted tongue tip	0.21	0.44
		2	Backed tongue body	1.61	1.94
		3	Lowered tongue body	1.34	1.71
		4	Pharyngeal constriction	0.90	1.18
		5	Nasal	0.11	0.24
	Muscular tension	7	Tense vocal tract	0.27	0.43
		8	Lax larynx	0.06	0.19
	Phonation	9	Whisper	0.32	0.44
		10	Harsh voice	0.99	1.32
		11	Short‐term events—pauses	0.26	0.38
Control group	Vocal tract	12	Lips—extensive range	0.31	0.85
		13	Jaw—minimized range	0.32	1.39
		14	Lowered tongue body	0.91	1.39
	Phonation	15	Voice	0.37	0.72
		16	Whispery voice	0.41	0.90

Abbreviations: IL, inferior limit; SL, superior limit.

The discriminant analysis demonstrated high accuracy in differentiating the groups, with 98.68% of RG samples and 98.31% of CG samples correctly classified based on perceptual voice quality judgments. This classification was achieved using a single discriminant function, indicating that the examined perceptual variables were sufficiently robust to distinguish between the two groups with minimal overlap.

The discriminant function was a linear combination of VQS, with several contributing significantly to group separation. The most influential variables included backed tongue body, pharyngeal constriction, and lowered tongue body, each contributing over 45% to the discriminant model. Additional relevant features included laryngeal height (raised larynx), lip range, and phonatory qualities such as a harsh voice, a creaky voice, and varying degrees of whisper. Collectively, these variables constituted a perceptual profile capable of reliably differentiating individuals with AIDS from those in the CG (Table 3).

**TABLE 3:** Influential voice quality settings for the discrimination factor of perceptual judgments of voice quality and their influence percentages.

Voice quality settings	Influence (%)
Backed tongue body	49.5
Pharyngeal constriction	46.8
Lowered tongue body	46.2
Raised larynx	40.8
Extensive lip range	37.7
Harsh voice	35.0
Creaky voice	34.8
Whispery voice	31.7
Whisper	31.4
Tense vocal tract	23.6

The discriminant function based on perceptual variables demonstrated high classification accuracy for speaker gender. The samples were separated by a single discriminant factor, comprising a specific combination of VQS that effectively distinguished male and female voices with substantial precision. When considered individually, the data indicated that 99.82% of speaker emissions were accurately classified. Only one participant, number 8 from the CG, exhibited a slightly lower identification rate, with 93.75% of emissions correctly attributed. All other speakers were classified without error. Regarding gender classification, perceptual data yielded correct estimation rates of 95.62% for female speakers and 98.37% for male speakers. These results underscore the robustness of the discriminant model, which is supported by a set of VQS that reflect articulatory and phonatory characteristics consistently associated with speaker gender (Table 4).

**TABLE 4:** Influential voice quality settings for the discrimination factor of speaker gender and their influence percentages.

Voice quality settings	Influence (%)
Backed tongue body	51.2
Labiodentalization	42.2
Advanced tongue tip	40.7
Lowered tongue body	40.5
Harsh voice	28.9
Lax vocal tract	28.3
Lowered tongue body	27.7
Minimized tongue body range	27.4
Denasal	20.5
Creaky voice	20.4

Within the gender subgroups of the RG, the perceptual data indicated a high level of classification accuracy, with 94.29% for female voices and 99.17% for male voices. A single discriminant factor, derived from a combination of VQS and their respective influence levels, was sufficient to separate the samples (Table 5). The most influential variables were backed tongue body and labiodentalization, each contributing over 50% to the discriminant model. The presence of modal voice and advanced tongue tip also played significant roles in distinguishing between genders within the RG, further demonstrating the effectiveness of the discriminant function applied to this subgroup of the sample.

**TABLE 5:** Influential voice quality settings for the discrimination factor of speaker gender and their influence percentages at subgroups of RG speakers.

Voice quality settings	Influence (%)
Backed tongue body	53.3
Labiodentalization	51.6
Voice	50.2
Advanced tongue tip	42.0

In the gender‐based subgroups of the CG, perceptual data indicated 100% accuracy in classifying both female and male speakers. The samples were distinguished by a single discriminant factor, consisting of a specific combination of VQS (Table 6). The most influential VQS were lowered tongue body, minimized jaw range, and minimized tongue body range, all of which exhibited high discriminatory power. The presence of voice breaks also contributed significantly to the model, indicating that these articulatory and phonatory features were sufficient to differentiate speaker gender within the CG reliably.

**TABLE 6:** Influential voice quality settings for the discrimination factor of speaker gender and their influence percentages at subgroups of CG speakers.

Voice quality settings	Influence (%)
Lowered tongue body	69.1
Minimized jaw range	61.2
Minimized tongue body range	59.7
Voice breaks	58.3

### 3.2 Acoustic Data: Measurements Extracted From the Expression Evaluator Script

Analysis of acoustic measurements from speaker emissions in the RG and the CG revealed statistically significant differences in intensity asymmetry and the standard deviation of the LTAS, as confirmed by hypothesis testing. Discriminant analysis demonstrated that acoustic variables were capable of partially distinguishing between the two groups, correctly classifying 77.37% of RG samples and 77.53% of CG samples. A single discriminant factor accounted for this separation, combining several acoustic measures, each contributing a distinct percentage of influence (Table 7). Among the most relevant measures were intensity asymmetry and spectral slope variability, followed by pitch‐related parameters and spectral slope asymmetry. These findings suggest that subtle variations in vocal intensity and frequency distribution contribute meaningfully to distinguishing vocal emissions between individuals living with AIDS and those in the CG.

**TABLE 7:** Influential acoustic measures for the discriminant factor between RG and CG speakers and their respective influence percentages.

Acoustic measures	Influence (%)
Intensity—asymmetry	55.1
Spectral slope—standard deviation	51.2
f0 ‐ Semiamplitude between quartiles	40.0
Spectral slope—mean	38.0
Spectral slope—asymmetry	34.8
f0 ‐ Median	28.3

When analysed at the individual level, the acoustic data demonstrated an overall classification accuracy of 84.77% across speakers. Only five speakers achieved complete discrimination, with just one from the CG. The remaining participants exhibited discrimination rates of 33.33–96.67%, indicating variable performance in acoustic differentiation at the individual level. In contrast, the analysis based on speaker gender revealed complete classification accuracy. The acoustic data obtained using the Expression Evaluator script enabled 100% correct identification of both male and female subgroups. This high level of discrimination was achieved through a single discriminant factor, composed of a specific combination of acoustic parameters (Table 8). The most influential variable was the semiamplitude between quartiles of the fundamental frequency (f0), which contributed fully to the model. The median f0 and the asymmetry of the spectral slope also played supporting roles in distinguishing vocal emissions by gender.

**TABLE 8:** Influential acoustic measures for the discrimination factor of speaker gender and their respective influence percentages.

Acoustic measures	Influence (%)
f0 ‐ Semiamplitude between quartiles	100.0
f0 ‐ Median	54.2
Spectral slope—asymmetry	31.5

Regarding the acoustic measures derived from the Expression Evaluator script, analysis of gender‐based subgroups within the RG revealed complete accuracy in speaker classification. The data demonstrated a 100% success rate in estimating both female and male voices. This result was achieved through a single discriminant factor, constructed from a specific combination of acoustic variables (Table 9). The most influential parameter was the semiamplitude between quartiles of the f0, which alone accounted for the entire discrimination capacity. Additional contributions were made by the median f0 and the standard deviation of the spectral slope, both of which enhanced the discriminatory power in distinguishing gender‐related vocal patterns among RG participants.

**TABLE 9:** Influential acoustic measures for the discrimination factor of speaker gender and their influence percentages within RG speaker subgroups.

Acoustic measures	Influence (%)
f0 ‐ Semiamplitude between quartiles	100.0
f0 ‐ Median	59.7
Spectral slope—standard deviation	32.8

In the gender‐based subgroups of the CG, the acoustic measurements obtained from the Expression Evaluator script yielded perfect classification accuracy. The model accurately classified both female and male speakers. This discrimination was achieved using a single factor, a specific combination of acoustic parameters, with their respective influence values presented in Table 10. The f0 semiamplitude between quartiles was the most decisive variable, contributing nearly all of the model's discriminative power. The standard deviation of the LTAS and the mean spectral slope also made significant contributions, collectively reinforcing the acoustic distinction of speaker gender within the CG subgroup.

**TABLE 10:** Influential acoustic measures for the discrimination factor of speaker gender and their influence percentages at subgroups of CG speakers.

Acoustic measures	Influence (%)
f0 ‐ Semiamplitude between quartiles	99.9
LTAS—Standard deviation	79.0
Spectral slope—mean	41.4

The linear regression analysis of the acoustic measurements extracted by the ExpressionEvaluator script indicated a correlation coefficient (*R* ^2^) of only 36.5% for the scoring groups (RG and CG). Thus, the data account for only 36.5% of the variance, while 63.5% is attributable to random or unknown.

## 4 Discussion

Various communication disorders have been reported among people living with AIDS; however, these issues have not been adequately addressed in the literature, either in terms of exploring their underlying nature or in proposing interventions to facilitate treatment and establish preventive strategies based on the identification of speech biomarkers. In this context, phonetic (perceptual) analysis of voice quality separated speakers into RGs and CGs with nearly 100% accuracy (Camargo and Madureira 2008).

The discriminant analysis of speaker groups using perceptual data demonstrated a greater influence of vocal tract VQS (backed and lowered tongue body, pharyngeal constriction, and raised larynx), followed by phonation VQS (a combination of harsh voice with whisper and creaky voice), and, lastly, muscular tension VQS (tense vocal tract and lax larynx). The acoustic data showed lower discriminative power than the perceptual data, with separation rates of 77.37% for the RG and 77.53% for the CG. Hence, this discussion addresses the acoustic aspects of their integration with perceptual data, focusing on correlations between perceptual and potential acoustic biomarkers of voice quality.

Clinically, the correlation between perceptual‐auditory and acoustic indices is relevant, as it integrates multidimensional clinical interpretation of voice quality with objective, reproducible measures (Camargo and Madureira 2009; Laver et al. 1981). While the perceptual analyses demonstrated greater discriminative power than the acoustic measures alone, the relationship between perceptual VQS and their acoustic correlates provided a more comprehensive understanding of the functional adjustments of the vocal tract. These findings reinforce the clinical value of combining perceptual and acoustic analyses for the functional characterization of the vocal tract and support the investigation of potential voice biomarkers during the longitudinal follow‐up in people living with AIDS (Barche et al. 2020; Chandrashekar et al. 2020).

From a perceptual perspective, the VQS of pharyngeal constriction and backed tongue body were prominent in the RG. Pharyngeal constriction leads to the closure of the middle oropharynx via contraction of the pharyngeal walls and/or retraction of the tongue dorsum (Aihara et al. 2021). Anatomical alterations of the oral and pharyngeal cavities, as well as the larynx, have been documented in the AIDS population, particularly as a result of Kaposi's Sarcoma (Alves et al. 2021; Moorad et al. 2023). These findings regarding VQS of the pharyngeal cavity and tongue posture underscore the importance of a functional approach to this region for generating vocal signals in the studied population. They also highlight the relevance of other head and neck functions, such as breathing and swallowing.

When analysing differences by gender subgroups, the following VQS were identified for females: minimized tongue body and jaw ranges, raised larynx, creaky voice, and short‐term episodes of diplophonia; for males: retracted tongue tip, lowered tongue body, tense vocal tract, lax larynx, whisper, and harsh voice. In the female RG subgroup, VQS related to minimized jaw and tongue ranges may be supported by evidence of lipodystrophy in the cervical and facial regions, which could explain decreased ranges of articulator movement (Alves et al. 2014; Finkelstein et al. 2015). The tendency to combine a creaky voice with short‐term episodes of diplophonia suggests irregularities in vocal fold vibratory activity, indicating potential integration between vocal tract and phonatory mechanisms (Camargo and Madureira 2009).

In the male RG subgroup, vocal tract VQS (retracted tongue tip, lowered tongue body) may be associated with the previously mentioned oral and pharyngeal changes. In fact, researchers have described manifestations such as reduced tongue projection and hypo‐ or hypertonia of the lips and tongue in this population (Bartholo et al. 2023; Dominguez Filho et al. 2021). In the phonatory system, the combination of whisper and harsh voice VQS indicates an association of factors responsible for the pathophysiology of the voice, which corroborates the concept of “hoarseness” as a composite VQS, corresponding to references to laryngeal disorders in this population (Badji et al. 2022; Brandão Canut et al. 2021). For phonatory (laryngeal) VQS, such a combination of settings indicates a voice disorder, which may be associated with the presence of opportunistic diseases affecting phonatory system structures and causing muscular hypotonia, with hoarseness being the main complaint (Badji et al. 2022; Sanjar et al. 2011; Watson et al. 2004). Similarly, lipodystrophy in the vocal folds could account for laryngeal hypotonia (Hultman et al. 2007; Mallewa et al. 2008).

Integrated statistical analysis of perceptual and acoustic data revealed the strongest correlations between vocal tract, muscular tension, and phonatory VQS with acoustic measures of LTAS, spectral slope, and f0. The lower discriminative power of acoustic analysis (*R* ^2^ = 36.5%) compared to perceptual evaluation may be explained by the nature of the parameters involved. The acoustic features extracted using the Expression Evaluator script in Praat are isolated and quantitative, whereas perceptual analysis captures multidimensional and integrative aspects of voice quality. Furthermore, speaker variability and the nature of the speech tasks (read vs. semi‐spontaneous) may influence acoustic consistency. Nonetheless, the acoustic parameters were selected based on expressiveness‐oriented principles (Barbosa 2009), which may not fully reflect the complexity perceived in human auditory processing.

In this study, data from the male subgroup of the RG revealed the relevance of the VQS for laryngeal hypofunction, consistent with findings from LTAS standard deviation acoustic measurements. Notably, differences in acoustic measurements between the RG and CG were observed only in the male subgroup. Overall, in the group of speakers with AIDS, VQS trends related to the vocal tract, phonation, and muscular tension support the relationship between frequent infections of the oral cavity, pharynx, and larynx and their influence on speech and voice instability in people living with AIDS (Ndjolo et al. 2004).

The phonetic description of voice quality provides detailed insights into perceptual voice assessment by characterizing the activity of speech and voice mechanisms (Laver et al. 1981). Given the nature of the perceptual findings and the limited sensitivity of the selected acoustic measures to describe and characterize the voice quality profiles of male and female subgroups in the RG and CG, future research should include complementary acoustic analyses that capture supralaryngeal vocal tract features and muscular tension. Exploration of formant patterns is a promising avenue. It is noteworthy that LTAS (standard deviation), spectral decline (mean and standard deviation), and f0 (amplitude between quartiles, median, and 99.5% quantile) measurements were relevant when considered alongside perceptual voice quality profile data.

Anatomical changes caused by abnormal body fat distribution can be classified in the head and neck region as lipoatrophy (loss of peripheral fat) and lipohypertrophy (accumulation of central and/or localized fat in the cervical region or neck). As for lipodystrophy, loss of facial fat and accumulation of fat in the neck may affect settings in both the supralaryngeal (articulatory) and laryngeal (phonatory) planes, which are responsible for voice quality. These alterations impact the vertical dimension of the vocal tract (raised larynx), its diameter (retracted tongue and pharyngeal constriction), and features related to the velopharyngeal segment (nasal and denasal) (Laver et al. 1981).

These findings suggest a relationship between vocal characteristics and antiretroviral medication among RG members, particularly when considering the chronic degenerative manifestations in the structures comprising the phonatory apparatus. Based on the VQS related to the phonatory apparatus in RG participants, future research should include physiological studies of the different segments of the speech and voice mechanism (supralaryngeal vocal tract, larynx, and muscle system) to provide insights into the anatomical‐physiological or pathophysiological conditions described above.

Despite our promising findings, several limitations must be acknowledged. The results must be interpreted within the clinical complexity of older people living with AIDS. Rather than isolating the individual contribution of each comorbidity to voice quality, this study aimed to characterize the voice quality profile of individuals undergoing long‐term follow‐up in a tertiary reference service of the Brazilian Unified Health System (Alves et al. 2023; DST/Aids 2018). Additionally, participants were recruited from the same specialized service, within the same age range, and socio‐phonetic variables were considered in the statistical analysis. Therefore, comorbidities were not regarded as external confounding factors but as part of the clinical complexity that characterizes this population, together with aging, long‐term antiretroviral therapy, lipodystrophy, and opportunistic diseases (Lamesa 2024). Consequently, the observed voice quality patterns should be interpreted as reflecting the combined functional consequences of these conditions rather than the isolated effect of a single clinical factor.

Aging leads to structural modifications in the vocal tract, including atrophy of the laryngeal musculature and ossification of cartilaginous structures. Such changes contribute to the development of presbyphonia and increase susceptibility to dysphonia (Rachid and Schechter 2017; Sataloff and Kost 2020). Classical risk factors such as smoking, excessive alcohol consumption, and vocal overuse are well recognized due to their high prevalence and clinical detectability. Nevertheless, in people with AIDS, the increased incidence of laryngeal disorders is also associated with chronic immunosuppression and opportunistic diseases, both of which negatively impact vocal quality (Piersiala et al. 2022). Many older adults, however, interpret these vocal changes as a natural consequence of aging, contributing to underreporting of symptoms and delays in accessing appropriate clinical intervention (Beton et al. 2022).

As follow‐up care for the AIDS population and their contacts evolves within the Brazilian health system, understanding the specific characteristics of these groups contributes to expanding health care strategies and preventing or minimizing the consequences of the disease and its treatments. In this sense, perceptual‐auditory and acoustic characterization of voice quality may help better identify functional alterations of the vocal tract, supporting longitudinal monitoring and future preventive and rehabilitative speech‐language pathology strategies.

The main clinical contribution of this study lies in providing a clinically grounded description of voice quality patterns in older adults living with AIDS. Although the findings do not support the proposal of an immediate therapeutic protocol, they may support clinical reasoning and future speech and language pathology decision‐making by indicating which perceptual and acoustic dimensions should be considered during assessment and follow‐up.

One methodological limitation of this study concerns the discrepancy in sample sizes between the RG (*n* = 20) and the CG (*n* = 9), with the RG comprising individuals with AIDS and the CG comprising individuals without AIDS. This asymmetry was due to the stringent inclusion criteria for the CG, which required participants to be within the same age range (50–60 years), free from HIV infection, antiretroviral therapy, and metabolic alterations such as dyslipidaemia, and without any self‐reported history of speech or voice disorders.

These criteria were essential to ensure clinical homogeneity and the internal validity of the comparisons despite making recruitment particularly challenging, especially given the limited availability of individuals meeting all conditions in this age group. The perceptual evaluation achieved high classification accuracy (98.68% for the RG and 98.31% for the CG), indicating that unequal group sizes did not compromise the discriminative power of the analysis. Nonetheless, this limitation is acknowledged as a potential limitation on the generalizability of the findings, and future studies should aim for larger and more balanced samples to confirm and expand upon these findings.

## 5 Conclusion

The integrated analysis of perceptual and acoustic data revealed strong correlations between perceptual VQS involving the vocal tract and muscle tension and acoustic measures of LTAS, spectral slope, and f0. These findings may reflect the combined functional consequences of long‐term antiretroviral therapy, opportunistic diseases, lipodystrophy, and associated metabolic alterations. Despite its descriptive nature, this study does not allow for the immediate proposal of a therapeutic protocol, as the results provide a clinical foundation for early identification of voice quality changes, longitudinal monitoring, and future speech‐language pathology decision‐making. This underscores the need for further research to expand the description of these phenomena and support the future development of preventive, monitoring, and rehabilitative speech‐language pathology strategies for the long‐term follow‐up care of older people living with AIDS.

## Funding

This research was supported by the National Council for Scientific and Technological Development (CNPq) under Grant No. 311596/2022‐23.

## Ethics Statement

The research project was approved by the Research Ethics Committee of the Emílio Ribas Institute of Infectious Diseases, São Paulo Municipal Health Department—Unified Health System (SUS)—Ministry of Health, Brazilian Government, under research protocol No. 71/2011.

## Consent

The participants in the study signed the Free and Informed Consent Form, expressing their agreement to the use of their information for strictly scientific purposes.

## Conflicts of Interest

The authors declare no conflict of interest.

## Supporting information




**Supporting Information**: jlcd70309‐supp‐0001‐SuppMat.docx

## Data Availability

Data generated during this study are available from the corresponding author upon reasonable request.

## References

[jlcd70309-bib-0002] Aihara, K. , Y. Inamoto , D. Kanamori , et al. 2021. “Effect of Tongue‐Hold Swallow on Posterior Pharyngeal Wall Using Dynamic Area Detector Computed Tomography.” Journal of Oral Rehabilitation 48, no. 11: 1235–1242. 10.1111/joor.13246. 34407238 PMC9291453

[jlcd70309-bib-0003] Alves, A. M. , A. C. D. Santos , A. Kumow , A. P. S. Sato , E. T. S. Helena , and M. I. B. Nemes . 2023. “Para Além do Acesso Ao Medicamento: papel do SUS e Perfil da Assistência em HIV no Brasil.” Revista de Saúde Pública 57: 26. 10.11606/s1518-8787.2023057004476. 37075422 PMC10118421

[jlcd70309-bib-0004] Alves, C. G. B. , M. S. Assis , A. D. S. Maciel , et al. 2021. “Clinical and Laboratory Profile of People Living with HIV/AIDS with Oral Kaposi Sarcoma.” AIDS Research and Human Retroviruses 37, no. 11: 870–877. 10.1089/AID.2020.0311. 34538064

[jlcd70309-bib-0005] Alves, M. D. , C. Brites , and E. Sprinz . 2014. “HIV‐Associated Lipodystrophy: A Review from a Brazilian Perspective.” Therapeutics and Clinical Risk Management 10: 559–566. 25083134 10.2147/TCRM.S35075 PMC4108257

[jlcd70309-bib-0006] Askari, S. , L. Fellows , M. J. Brouillette , and N. Mayo . 2021. “Development and Validation of a Voice‐of‐the‐Patient Measure of Cognitive Concerns Experienced by People Living with HIV.” Quality of Life Research 30: 921–930. 10.1007/s11136-020-02679-z. 33104940

[jlcd70309-bib-0007] Badji, A. , Y. Dieng , I. Diop , P. A. Cisse , and B. Diouf . 2022. “State‐of‐the‐Art of the Impact of HIV and Its Treatment on the Voice of PLHIV.” In Proceedings of Sixth International Congress on Information and Communication Technology: ICICT 2021 London 1: 137–146. 10.1007/978-981-16-2377-6_15.

[jlcd70309-bib-0008] Baldi de Freitas, A. 2017. “Voice Quality and Acromegaly: Acoustics and Perception.” Intercâmbio 34: 1–16. http://revistas.pucsp.br/index.php/intercambio/article/view/33519.

[jlcd70309-bib-0009] Barbosa, P. A. 2009. “Detecting Changes in Speech Expressiveness in Participants of a Radio Program.” In INTERSPEECH 2009 : 2155–2158.

[jlcd70309-bib-0010] Barche, P. , K. Gurugubelli , and A. K. Vuppala . 2020. “Towards Automatic Assessment of Voice Disorders: A Clinical Approach.” In INTERSPEECH 2020 : 2537–2541. 10.21437/Interspeech.2020-2160.

[jlcd70309-bib-0011] Bartholo, M. F. , J. R. Tenório , N. S. Andrade , P. P. Shibutani , F. Martins , and M. Gallottini . 2023. “Orofacial Manifestations in Brazilian People Living with HIV/AIDS Under Long‐Term Antiretroviral Therapy: A Cross‐Sectional Study.” Oral Surgery, Oral Medicine, Oral Pathology and Oral Radiology 136, no. 4: 436–441. 10.1016/j.oooo.2023.05.001. 37271609

[jlcd70309-bib-0012] Beton, S.ü , L. Yücel , H. Başak , and Z. Çiler Büyükatalay . 2022. “The Elderly Voice: Mechanisms, Disorders and Treatment Methods.” Turkish Archives of Otorhinolaryngology 60, no. 4: 220–226. 10.4274/tao.2022.2022-8-1. 37456599 PMC10339270

[jlcd70309-bib-0013] Boersma, P. , and D. Weenink . 2018. Praat: doing phonetics by computer. Version 6.0.37.

[jlcd70309-bib-0014] Brandão Canut, M. S. , G. C. Pereira , A. P. Silva , L. R. Santos , and T. F. Oliveira . 2021. “Achados Fonoaudiológicos na Síndrome da Imunodeficiência Adquirida: Revisão Integrativa.” Saúde Coletiva 11, no. 62: 5272–5281. 10.36489/saudecoletiva.2021v11i62p5272-5281.

[jlcd70309-bib-0015] Camargo, Z. , and P. Canton . 2020. “Vocal Quality of Children with Altered Frenulum in the Tongue.” Journal of Speech Sciences 8, no. 1: 15–26. 10.20396/joss.v8i1.14990.

[jlcd70309-bib-0016] Camargo, Z. , and S. Madureira . 2008. “Voice Quality Analysis from a Phonetic Perspective: Voice Profile Analysis Scheme (VPAS) Profile for Brazilian Portuguese.” In: Proceedings of the 4th International Conference on Speech Prosody 2008 : 57–60. 10.21437/SpeechProsody.2008-13.

[jlcd70309-bib-0017] Camargo, Z. , and A. Rilliard . 2019. “Interactions of Voice Quality Settings.” In: *Proceedings of the 10th International Conference of Experimental Linguistic*s (ExLing 2019) *2019* : 42–44. 10.36505/ExLing-2019/10/0010/000372.

[jlcd70309-bib-0018] Camargo, Z. A. , and S. Madureira . 2009. “Dimensões Perceptivas das Alterações de Qualidade Vocal e Suas Correlações aos Planos Da Acústica e da Fisiologia.” DELTA: Documentação de Estudos em Lingüística Teórica e Aplicada 25, no. 2: 285–317. 10.1590/S0102-44502009000200004.

[jlcd70309-bib-0056] Chandrashekar, H. M. , V. Karjigi , and N. Sreedevi . 2019. “Breathiness Indices for Classification of Dysarthria Based on Type and Speech Intelligibility.” In 2019 International Conference on Wireless Communications, Signal Processing and Networking (WiSPNET). IEEE. 10.1109/WiSPNET45539.2019.9032852.

[jlcd70309-bib-0055] Chandrashekar, H. M. , V. Karjigi , and N. Sreedevi . 2020. “Spectro‐Temporal Representation of Speech for Intelligibility Assessment of Dysarthria.” IEEE Journal of Selected Topics in Signal Processing 14, no. 2: 390–399. 10.1109/JSTSP.2019.2949912.

[jlcd70309-bib-0020] Chawla, A. , C. Wang , C. Patton , et al. 2018. “A Review of Long‐Term Toxicity of Antiretroviral Treatment Regimens and Implications for an Aging Population.” Infectious Diseases and Therapy 2: 183–195. 10.1007/s40121-018-0201-6. PMC5986685 29761330

[jlcd70309-bib-0021] Condori, R. P. , N. Reis , and Z. Camargo . 2025. “Percepção Auditiva do Contraste de Vozeamento em Falantes Laringectomizados Totais.” Audiology—Communication Research 30: e3008. 10.1590/2317-6431-2024-3008pt.

[jlcd70309-bib-0022] de Souza Fonseca, R. R. , R. V. Laurentino , S. A. F. de Menezes , et al. 2022. “Digital Form for Assessing Dentistsʼ Knowledge About Oral Care of People Living with HIV.” International Journal of Environmental Research and Public Health 19, no. 9: 5055. 10.3390/ijerph19095055. 35564449 PMC9103845

[jlcd70309-bib-0023] Dominguez Filho, O. J. L. , E. C. Viana , W. G. Pessoa , and P. R. C. Domingos . 2021. “Manifestações Orais em Pacientes Imunodeprimidos pelo Vírus da Imunodeficiência Humana (HIV): Revisão Da Literatura.” Revista Eletrônica Acervo Saúde 13, no. 2: e6034. 10.25248/reas.e6034.2021.

[jlcd70309-bib-0024] DST/Aids, C. 2018. “Assistência às Pessoas com IST e Vivendo com HIV e aids no Estado de São Paulo.” Boletim Epidemiológico Paulista 15, no. 175/176: 37–59. 10.57148/bepa.2018.v.15.39179.

[jlcd70309-bib-0025] Fernandes, A. C. N. , Z. Camargo , S. P. P. Giannini , L. C. Rusilo , and L. P. Ferreira . 2025. “Phonetic Description of the Voice of Teachers From Public Schools in São Paulo.” Distúrbios da Comunicação 37, no. 2: e68945. 10.23925/2176-2724.2025v37i2e68945.

[jlcd70309-bib-0026] Finkelstein, J. L. , P. Gala , R. Rochford , M. J. Glesby , and S. Mehta . 2015. “HIV/AIDS and Lipodystrophy: Implications for Clinical Management in Resource‐Limited Settings.” Journal of the International AIDS Society 18, no. 1: 19033. 10.7448/IAS.18.1.19033. 25598476 PMC4297925

[jlcd70309-bib-0027] Fisicaro, V. , E. Campanella , A. Marino , et al. 2023. “Laryngeal Leishmaniasis in a HIV‑Positive Patient: A Case Report and Review of the Literature.” World Academy of Sciences Journal 5: 24. 10.3892/wasj.2023.201.

[jlcd70309-bib-0028] Furrer, H. 2016. “Opportunistic Diseases during HIV Infection—Things Aren′T What They Used to Be, or Are They?” Journal of Infectious Diseases 214, no. 6: 830–831. 10.1093/infdis/jiw086. 27559121

[jlcd70309-bib-0029] Haase, K. , I. Piwonski , C. Stromberger , et al. 2021. “Incidence and Survival of HNSCC Patients Living With HIV Compared with HIV‐Negative HNSCC Patients.” European Archives of Oto‐Rhino‐Laryngology 278, no. 10: 3941–3953. 10.1007/s00405-020-06573-9. 33492419 PMC8382606

[jlcd70309-bib-0031] Hultman, C. S. , L. E. McPhail , J. H. Donaldson , and D. A. Wohl . 2007. “Surgical Management of HIV‐Associated Lipodystrophy.” Annals of Plastic Surgery 58, no. 3: 255–263. 10.1097/01.sap.0000248128.33465.83. 17471128

[jlcd70309-bib-0032] Ivanova, G. , R. Alhrahsheh , and K. Kukov . 2023. “Cognitive Deficits: Verbal and Semantic Fluency in People Living with HIV and AIDS.” Current HIV Research 21, no. 3: 202–212. 10.2174/1570162;21666230613124. 37312442 PMC10556403

[jlcd70309-bib-0033] Jesus, T. V. , A. N. P. Almeida , and Z. A. Camargo . 2021. “Ultrasonography Applied to the Description of Voice Quality Settings in Adult Speakers of Brazilian Portuguese.” Revista CEFAC 23, no. 6: e4921. 10.1590/1982-0216/20212364921.

[jlcd70309-bib-0034] Jordan, R. E. , P. Adab , and K. K. Cheng . 2020. “Covid‐19: Risk Factors for Severe Disease and Death.” Bmj 368: 1–2. 10.1136/bmj.m1198. 32217618

[jlcd70309-bib-0035] Lamesa, T. A. 2024. “Biological Depiction of Lipodystrophy and its Associated Challenges Among HIV AIDS Patients: Literature Review.” HIV/AIDS—Research and Palliative Care 16: 123–132. 10.2147/HIV.S445605. 38584795 PMC10999207

[jlcd70309-bib-0036] Laver, J. , S. L. Wirz , J. Mackenzie‐Beck , and S. M. Hiller . 1981. “A Perceptual Protocol for the Analysis of Vocal Profiles.” Work in Progress—Department of Linguistics, University of Edinburgh 14: 139–155.

[jlcd70309-bib-0058] Laver, J. 1980. The Phonetic Description of Voice Quality. Cambridge: Cambridge University Press. ISBN 0‐521‐23176‐0.

[jlcd70309-bib-0037] Lomelí‐Martínez, S. M. , L. A. González‐Hernández , A. J. Ruiz‐Anaya , et al. 2022. “Oral Manifestations Associated with HIV/AIDS Patients.” Medicina 58, no. 9: 1214. 10.3390/medicina58091214. 36143891 PMC9504409

[jlcd70309-bib-0038] Mallewa, J. E. , E. Wilkins , J. Vilar , et al. 2008. “HIV‐Associated Lipodystrophy: A Review of Underlying Mechanisms and Therapeutic Options.” Journal of Antimicrobial Chemotherapy 62, no. 4: 648–660. 10.1093/jac/dkn251. 18565973

[jlcd70309-bib-0039] McLaurin, K. A. , M. Harris , V. Madormo , S. B. Harrod , C. F. Mactutus , and R. M. Booze . 2021. “HIV‐Associated Apathy/Depression and Neurocognitive Impairments Reflect Persistent Dopamine Deficits.” Cells 10, no. 8: 2158. 10.3390/cells10082158. 34440928 PMC8392364

[jlcd70309-bib-0040] Ministério da Saúde . 2025. Secretaria de Vigilância em Saúde e Ambiente. Departamento de HIV, Aids, Tuberculose, Hepatites Virais e Infecções Sexualmente Transmissíveis. Boletim Epidemiológico: HIV e Aids 2025. Número Especial, dezembro de 2025. Ministério da Saúde.

[jlcd70309-bib-0041] Moorad, R. , E. Kasonkanji , J. Gumulira , et al. 2023. “Manifestations Oto‐rhino‐laryngologiques Inaugurales de L'infection à VIH/SIDA: Analyse de 76 cas Observés en Milieu Africain [Early ENT manifestations of HIV infection/AIDS: An Analysis of 76 Cases Observed in Africa].” Revue de Laryngologie—Otologie—Rhinologie 125, no. 1: 39–43. 10.1002/ijc.34689. 15244028

[jlcd70309-bib-0042] Ndjolo, A. , R. Njock , N. M. Ngowe , et al. 2004. “Manifestations Oto‐Rhino‐Laryngologiques Inaugurales de L'infection à VIH/SIDA. Analyse de 76 cas Observés En Milieu Africain [Early ENT Manifestations of HIV Infection/AIDS. An Analysis of 76 Cases Observed in Africa].” Revue de Laryngologie, Otologie, Rhinologie 125, no. 1: 39–43. https://pubmed.ncbi.nlm.nih.gov/15244028/. 15244028

[jlcd70309-bib-0043] Obeagu, E. I. , and D. Nwosu . 2019. “Adverse Drug Reactions in HIV/AIDS Patients on Highly Active Antiretroviral Therapy: A Review of Prevalence.” International Journal of Current Research in Chemistry and Pharmaceutical Sciences 6, no. 12: 45–48. 10.22192/ijcrcps.2019.06.12.004.

[jlcd70309-bib-0044] Pessoa‐Almeida, A. N. , Z. Camargo , S. Madureira , and A. Meireles . 2014. “Prosodic Analysis of the Speech of a Child with Cochlear Implant.” In Proceedings of Speech Prosody 7: 1115–1118. 10.21437/SpeechProsody.2014-212.

[jlcd70309-bib-0045] Piersiala, K. , S. F. Weinreb , L. M. Akst , A. T. Hillel , and S. R. Best . 2022. “Laryngeal Disorders in People Living with HIV.” American Journal of Otolaryngology 43, no. 1: 103234. 10.1016/j.amjoto.2021.103234. 34560598

[jlcd70309-bib-0046] Rachid, M. , and M. Schechter . 2017. Manual De HIV/AIDS. Thieme Revinter Publicações LTDA.

[jlcd70309-bib-0047] Ripamonti, E. , and M. Clerici . 2021. “Living with Chronic HIV Disease in the Antiretroviral Era: The Impact of Neurocognitive Impairment on Everyday Life Functions.” Topics in Antiviral Medicine 29, no. 3: 386–396. https://pmc.ncbi.nlm.nih.gov/articles/PMC8384087. 34370420 PMC8384087

[jlcd70309-bib-0048] Sanjar, F. A. , B. E. U. P. Queiroz , and I. D. Miziara . 2011. “Otolaryngologic Manifestations in HIV Disease: Clinical Aspects and Treatment.” Brazilian Journal of Otorhinolaryngology 77, no. 3: 391–400. 10.1590/S1808-86942011000300020. 21739017 PMC9443745

[jlcd70309-bib-0049] San Segundo, E. , P. Foulkes , P. French , P. Harrison , V. Hughes , and C. Kavanagh . 2019. “The Use of the Vocal Profile Analysis for Speaker Characterization: Methodological Proposals.” Journal of the International Phonetic Association 49, no. 3: 353–378. 10.1017/S0025100318000130.

[jlcd70309-bib-0050] Sataloff, R. T. , and K. M. Kost . 2020. “The Effects of Age on the Voice, Part 1.” Journal of Singing 77, no. 1: 63–70.

[jlcd70309-bib-0051] Shanmuga Ashok, S. , and S. Rajendran . 2020. “A Study on Otorhinolaryngologic Manifestations in human Immunodeficiency Virus Infected Patients.” International Journal of Otorhinolaryngology and Head and Neck Surgery 6, no. 3: 471–477. 10.18203/issn.2454-5929.ijohns20200547.

[jlcd70309-bib-0052] Siedner, M. J. , and V. Triant . 2019. “Undetectable = Untransmittable and Your Health: The Personal Benefits of Early and Continuous Therapy for HIV Infection.” The Journal of Infectious Diseases 219: 173–176. 10.1093/infdis/jiy445. 30032272 PMC6306014

[jlcd70309-bib-0053] Silva, M. F. B. L. , S. Madureira , L. C. Rusilo , and Z. A. Camargo . 2017. “Vocal Quality Assessment: Methodological Approach for a Perceptive Data Analysis.” Revista CEFAC 19, no. 6: 831–841. 10.1590/1982-021620171961417.

[jlcd70309-bib-0057] Vazquez, J. A. 2000. “Therapeutic Options for the Management of Oropharyngeal and Esophageal Candidiasis in HIV/AIDS Patients.” HIV Clinical Trials 1, no. 1: 47–59. 10.1310/T7A7-1E63-2KA0-JKWD. 11590489

[jlcd70309-bib-0054] Watson, J. R. , D. Granoff , and R. Thayer Sataloff . 2004. “Dysphonia due to kaposi's Sarcoma as the Presenting Symptom of human Immunodeficiency Virus.” Journal of Voice 18, no. 3: 398–402. 10.1016/j.jvoice.2004.04. 15331114

