# Supplementary material for: Voice Quality in Individuals With Acquired Immunodeficiency Syndrome
Source: Int J Lang Commun Disord. 2026 Sep 2;61(5):e70309. doi: 10.1111/1460-6984.70309 (PMC13536515; doi:10.1111/1460-6984.70309)
Supplement: Supplementary file 1 [file JLCD-61-0-s001.docx]

**Appendix A**

**Figure 1:** *Vocal Profile Analysis Scheme – VPAS (2007)*

VPAS protocol (Laver, 1980)

Speaker: Date of recording: Judge: Recording ID:

|  | **First pass** | | **Second pass** | | | | | | | |
| --- | --- | --- | --- | --- | --- | --- | --- | --- | --- | --- |
|  | **Neutral** | **Non-neutral** | **Setting** | | **Moderate** | | | **Extreme** | | |
|  |  |  |  |  | **1** | **2** | **3** | **4** | **5** | **6** |
| **A. VOCAL TRACT FEATURES** | | | | | | | | | | |
| 1. Labial |  |  | Lip rounding/protrusion | |  |  |  |  |  |  |
|  |  |  | Lip spreading | |  |  |  |  |  |  |
|  |  |  | Labiodentalization | |  |  |  |  |  |  |
|  |  |  | Minimized range | |  |  |  |  |  |  |
|  |  |  | Extensive range | |  |  |  |  |  |  |
| 2. Mandibular |  |  | Close jaw | |  |  |  |  |  |  |
|  |  |  | Open jaw | |  |  |  |  |  |  |
|  |  |  | Protruded jaw | |  |  |  |  |  |  |
|  |  |  | Extensive range | |  |  |  |  |  |  |
|  |  |  | Minimized range | |  |  |  |  |  |  |
| 3. Lingual tip/blade |  |  | Advanced tip/blade | |  |  |  |  |  |  |
|  |  |  | Retracted tip/blade | |  |  |  |  |  |  |
| 4. Lingual body |  |  | Fronted tongue body | |  |  |  |  |  |  |
|  |  |  | Backed tongue body | |  |  |  |  |  |  |
|  |  |  | Raised tongue body | |  |  |  |  |  |  |
|  |  |  | Lowered tongue body | |  |  |  |  |  |  |
|  |  |  | Extensive range | |  |  |  |  |  |  |
|  |  |  | Minimized range | |  |  |  |  |  |  |
| 5. Pharyngeal |  |  | Pharyngeal constriction | |  |  |  |  |  |  |
|  |  |  | Pharyngeal expansion | |  |  |  |  |  |  |
| 6. Velopharyngeal |  |  | Audible nasal escape | |  |  |  |  |  |  |
|  |  |  | Nasal | |  |  |  |  |  |  |
|  |  |  | Denasal | |  |  |  |  |  |  |
| 7. Larynx height |  |  | Raised Larynx | |  |  |  |  |  |  |
|  |  |  | Lowered Larynx | |  |  |  |  |  |  |
| **B. OVERALL MUSCULAR TENSION** | | | | | | | | | | |
| 8. Vocal tract tension |  |  | Tense vocal tract | |  |  |  |  |  |  |
|  |  |  | Lax vocal tract | |  |  |  |  |  |  |
| 9. Laryngeal tension |  |  | Tense larynx | |  |  |  |  |  |  |
|  |  |  | Lax larynx | |  |  |  |  |  |  |
| **C. PHONATION FEATURES** | | | | | | | | | | |
|  | **SETTING** | | **Present** | | **Scalar Degree** | | | | | |
|  |  |  | **Neutral** | **Non-neutral** | **Moderate** | | | **Extreme** | | |
|  |  |  |  |  | **1** | **2** | **3** | **4** | **5** | **6** |
| 10. Voicing type | Modal Voice | |  |  |  | | | | | |
|  | Falsetto | |  |  |  | | | | | |
|  | Creak (vocal fry) | |  |  |  | | | | | |
|  | Creaky voice | |  |  |  |  |  |  |  |  |
| 11. Laryngeal frication | Air escape | |  |  |  | | | | | |
|  | Breathy voice | |  |  |  |  |  |  |  |  |
| 12. Laryngeal irregularity | Harsh voice | |  |  |  |  |  |  |  |  |

|  | | | **Neutral** | **Setting** | **Moderate** | | | | **Extreme** | | |
| --- | --- | --- | --- | --- | --- | --- | --- | --- | --- | --- | --- |
|  |  |  |  |  | **1** | **2** | **3** | **4** | | **5** | **6** |
| **D. PROSODIC FEATURES** | | | | | | | | | | | |
| 13. Pitch | Mean | |  | High |  |  |  |  | |  |  |
|  |  |  |  | Low |  |  |  |  | |  |  |
|  | Range | |  | Minimised range |  |  |  |  | |  |  |
|  |  |  |  | Extensive range |  |  |  |  | |  |  |
|  | Variability | |  | High |  |  |  |  | |  |  |
|  |  |  |  | Low |  |  |  |  | |  |  |
| 14. Loudness | Mean | |  | High |  |  |  |  | |  |  |
|  |  |  |  | Low |  |  |  |  | |  |  |
|  | Range | |  | Extensive range |  |  |  |  | |  |  |
|  |  |  |  | Minimized range |  |  |  |  | |  |  |
|  | Variability | |  | High |  |  |  |  | |  |  |
|  |  |  |  | Low |  |  |  |  | |  |  |
| **E. TEMPORAL ORGANIZATION** | | | | | | | | | | | |
| 15. Continuity | |  | | Interrupted |  |  |  |  | |  |  |
| 16. Rate | |  | | Fast |  |  |  |  | |  |  |
|  |  |  |  | Slow |  |  |  |  | |  |  |
| **F. OTHER FEATURES** | | | | | | | | | | | |
| 17. Respiratory support | | | | Adequate |  |  |  |  | |  |  |
|  |  |  |  | Inadequate |  |  |  |  | |  |  |
| 18. Dyplophonia | | | | Absent |  |  |  |  | |  |  |
|  |  |  |  | Present |  |  |  |  | |  |  |

*Vocal Profile Analysis Scheme – VPAS* (Laver, Mackenzie-Beck, 2007).

Nome: Data da gravação: Juiz: Identificação da gravação:

| **Qualidade vocal** | **Primeira passada** | | **Segunda passada** | | | | | | | | | | |
| --- | --- | --- | --- | --- | --- | --- | --- | --- | --- | --- | --- | --- | --- |
|  | **Neutro** | **Não neutro** | **Ajuste** | | **Moderado** | | | | **Extremo** | | | | |
|  |  |  |  |  | **1** | **2** | | **3** | **4** | **5** | | | **6** |
| **A. ELEMENTOS DO TRATO VOCAL** | | | | | | | | | | | | | |
| 1. Lábios |  |  | Arredondados/  protraídos | |  | |  |  |  | |  |  | |
|  |  |  | Estirados | |  | |  |  |  | |  |  | |
|  |  |  | Labiodentalização | |  | |  |  |  | |  |  | |
|  |  |  | Extensão diminuída | |  | |  |  |  | |  |  | |
|  |  |  | Extensão aumentada | |  | |  |  |  | |  |  | |
| 2. Mandíbula |  |  | Fechada | |  | |  |  |  | |  |  | |
|  |  |  | Aberta | |  | |  |  |  | |  |  | |
|  |  |  | Protraída | |  | |  |  |  | |  |  | |
|  |  |  | Extensão diminuída | |  | |  |  |  | |  |  | |
|  |  |  | Extensão aumentada | |  | |  |  |  | |  |  | |
| 3. Língua ponta/lâmina |  |  | Avançada | |  | |  |  |  | |  |  | |
|  |  |  | Recuada | |  | |  |  |  | |  |  | |
| 4. Corpo de língua |  |  | Avançado | |  | |  |  |  | |  |  | |
|  |  |  | Recuado | |  | |  |  |  | |  |  | |
|  |  |  | Elevado | |  | |  |  |  | |  |  | |
|  |  |  | Abaixado | |  | |  |  |  | |  |  | |
|  |  |  | Extensão diminuída | |  | |  |  |  | |  |  | |
|  |  |  | Extensão aumentada | |  | |  |  |  | |  |  | |
| 5. Faringe |  |  | Constrição | |  | |  |  |  | |  |  | |
|  |  |  | Expansão | |  | |  |  |  | |  |  | |
| 6. Velofaringe |  |  | Escape nasal audível | |  | |  |  |  | |  |  | |
|  |  |  | Nasal | |  | |  |  |  | |  |  | |
|  |  |  | Denasal | |  | |  |  |  | |  |  | |
| 7. Altura de laringe |  |  | Elevada | |  | |  |  |  | |  |  | |
|  |  |  | Abaixada | |  | |  |  |  | |  |  | |
| **B. TENSÃO MUSCULAR GERAL** | | | | | | | | | | | | | |
| 8. Tensão do trato vocal |  |  | Hiperfunção | |  | |  |  |  | |  |  | |
|  |  |  | Hipofunção | |  | |  |  |  | |  |  | |
| 9. Tensão laríngea |  |  | Hiperfunção | |  | |  |  |  | |  |  | |
|  |  |  | Hipofunção | |  | |  |  |  | |  |  | |
| **C. ELEMENTOS FONATÓRIOS** | | | | | | | | | | | | | |
|  | **Ajuste** | | **Presente** | | **Graus de escala** | | | | | | | | |
|  |  |  | **Neutro** | **Não Neutro** | **Moderado** | | | | **Extremo** | | | | |
|  |  |  |  |  | **1** | | **2** | **3** | **4** | | **5** | **6** | |
| 10. Modo de fonação | Modal | |  |  |  | | | | | | | | |
|  | Falsete | |  |  |  | | | | | | | | |
|  | Crepitância/ *vocal fry* | |  |  |  | | | | | | | | |
|  | Voz crepitante | |  |  |  | |  |  |  | |  |  | |
| 11. Fricção laríngea | Escape de ar | |  |  |  | | | | | | | | |
|  | Voz soprosa | |  |  |  | |  |  |  | |  |  | |
| 12. Irregularidade laríngea | Voz áspera | |  |  |  | |  |  |  | |  |  | |

Ocorrências em curto termo ( ) quebras ( ) instabilidades ( ) diplofonia ( ) tremor. Para ajustes de ocorrência intermitente assinalar (i).

| **Dinâmica vocal** | | **Neutro** | **Ajuste** | **Moderado** | | | **Extremo** | | |
| --- | --- | --- | --- | --- | --- | --- | --- | --- | --- |
|  |  |  |  | **1** | **2** | **3** | **4** | **5** | **6** |
| **D. ELEMENTOS PROSÓDICOS** | | | | | | | | | |
| 13.*Pitch (f0)* | Habitual |  | Elevado |  |  |  |  |  |  |
|  |  |  | Abaixado |  |  |  |  |  |  |
|  | Extensão |  | Diminuída |  |  |  |  |  |  |
|  |  |  | Aumentada |  |  |  |  |  |  |
|  | Variabilidade |  | Diminuída |  |  |  |  |  |  |
|  |  |  | Aumentada |  |  |  |  |  |  |
| 14. *Loudness (intensidade)* | Habitual |  | Aumentado |  |  |  |  |  |  |
|  |  |  | Diminuído |  |  |  |  |  |  |
|  | Extensão |  | Diminuída |  |  |  |  |  |  |
|  |  |  | Aumentada |  |  |  |  |  |  |
|  | Variabilidade |  | Diminuída |  |  |  |  |  |  |
|  |  |  | Aumentada |  |  |  |  |  |  |
| 15. Tempo | | | | | | | | | |
| Continuidade | | | Interrompida |  |  |  |  |  |  |
| Taxa de elocução | | | Rápida |  |  |  |  |  |  |
|  |  |  | Lenta |  |  |  |  |  |  |
| 16. Outros elementos | | | | | | | | | |
| Suporte respiratório | | | Adequado |  |  |  |  |  |  |
|  |  |  | Inadequado |  |  |  |  |  |  |
|  |  |  | Presente |  |  |  |  |  |  |

Versão do *Vocal Profile Analysis Scheme – VPAS ou* Roteiro de avaliação da qualidade vocal com motivação fonética (Laver, Beck, 2007; adaptado por Camargo, 2007 revisado Camargo, 2002) – Camargo, Madureira, 2008.
